# Protracted Pregnancy

**Published:** 1854-10

**Authors:** 


Protracted Pregnancy.—Dr. Edward Smith mentions a case in which it appeared clear to him that the woman was delivered in ten months after conception took place. He also mentioned a case in which a child cried so loudly, three minutes after the head was born, and before the body was extruded, that it could be heard in the adjoining room. This case showed that when a pelvis was large, and the pains not severe, there might be sufficient room for the undelivered chest of the child to expand. Had there been a question in this case as to whether the child was born alive, it could have been shown that it had breathed. Dr. Smith also related a case in which an infant, two days after birth, had two of the incisors fully developed in its gums. In such cases, did the permanent teeth come earlier than in ordinary cases, and when teeth were developed in utero, were there three sets of teeth?

Mr. Charles Clark mentioned two cases in which pregnancy appeared to him to have been prolonged in one instance to ten, in the other to eleven months.

Mr. W. A. Harrison remarked, that in cases where teeth were developed so early as in Dr. Smith's case, they fell out early, the gums remaining bare for some time before the permanent teeth presented themselves, but the child had not three sets of teeth.

Mr. I. B. Brown and Mr. Dendy remarked that it was not rare to observe cases in which children cried before the shoulders were delivered.—*Lancet.*

